# Decrease of IL-5 Production by Naive T Cells Cocultured with IL-18-Producing BCG-Pulsed Dendritic Cells from Patients Allergic to House Dust Mite

**DOI:** 10.3390/vaccines9030277

**Published:** 2021-03-18

**Authors:** Magdalena Kowalewicz-Kulbat, Piotr Szpakowski, Krzysztof T. Krawczyk, Marek L. Kowalski, Slawomir Kosinski, Franck Biet, Wieslawa Rudnicka, Camille Locht

**Affiliations:** 1 Department of Immunology and Infectious Biology, Institute of Microbiology, Biotechnology and Immunology, Faculty of Biology and Environmental Protection, University of Lodz, 90-237 Lodz, Poland; piotr.szpakowski@umed.lodz.pl (P.S.); krzysztof.krawczyk@biol.uni.lodz.pl (K.T.K.); wieslawa.rudnicka@biol.uni.lodz.pl (W.R.); camille.locht@pasteur-lille.fr (C.L.); 2 Department of Neurology and Stroke, Medical University of Lodz, 90-549 Lodz, Poland; 3 Department of Immunology and Allergy, Medical University of Lodz, 92-213 Lodz, Poland; marek.kowalski@umed.lodz.pl; 4 Department of Immunology and Allergy, Central Clinical Hospital of Medical University of Lodz, 92-213 Lodz, Poland; kosinski@csk.umed.lodz.pl; 5 Institut National de Recherche pour l’Agriculture, l’Alimentation et l’Environnement (INRAE), Université de Tours, ISP, F-37390 Nouzilly, France; franck.biet@inrae.fr; 6 U1019–UMR9017–CIIL–Center for Infection and Immunity of Lille, Institut Pasteur de Lille, Université de Lille, CNRS, Inserm, CHU Lille, F-59000 Lille, France

**Keywords:** BCG, rBCG-hIL-18, dendritic cells, Th1/Th2 cells, asthma, Der p 1

## Abstract

The only currently available anti-tuberculosis vaccine, Bacillus Calmette–Guérin (BCG), has been reported to also protect against unrelated diseases, including inflammatory diseases such as allergic asthma. Recombinant BCG strains that produce IL-18 have been shown to enhance Th1 responses over non-recombinant BCG and to reduce IL-5 production and bronchoalveolar eosinophilia in mice. However, their ability to decrease the immune polarization of human Th2 cells is not known. Here, we show that BCG and recombinant BCG producing human IL-18 (rBCG-hIL-18) induced the maturation of Der p 1-stimulated monocyte-derived dendritic cells (MD-DCs) from healthy controls and from patients allergic to house dust mites. After incubation with mycobacteria and Der p 1, MD-DCs produced significantly more IL-23 and IP-10 but had no effect on IL-12p70 or IL-10 production compared to Der p 1-pulsed MD-DCs in the absence of mycobacteria. In the presence of Der p 1, BCG- and rBCG-hIL-18-pulsed MD-DCs cocultured with naive, but not with memory T cells from allergic patients, resulted in a decrease in IL-5 production compared to non-pulsed MD-DCs cultured in the presence of Der p 1. BCG, and especially rBCG-hIL-18, may thus be potential therapeutic tools to reduce exacerbated Th2 responses in patients with allergic asthma.

## 1. Introduction

Bacillus Calmette–Guérin (BCG), the only currently available vaccine against tuberculosis, has a number of beneficial off-target effects and is able to protect against various infectious and non-infectious diseases, including allergic asthma (for a review see [1]). Asthma is one of the most common forms of allergic disease in industrialized countries, and approximately 300 million people are affected by it. Although asthma is treatable, it continues to be a significant health burden and a major cause of disability-adjusted life span in adults and children [2]. Allergic asthma is characterized by excessive production of Th2-related cytokines by allergen-specific CD4^+^ T cells [3,4]. In contrast, IFN-γ-producing Th1 cells or IL-10 production by regulatory T cells are associated with the establishment of a healthy immune profile and tolerance induction in non-allergic subjects [4,5,6]. Allergen-specific CD4^+^ T cells exist in both allergic and non-allergic individuals, but they exhibit distinct cytokine profiles [6,7]. Cytokines produced by Th2 cells drive many features of asthma, such as IgE class switch, airway remodeling, airway hyperresponsiveness and mucus overproduction. They also play a central role in initiating and orchestrating the inflammatory response. House dust mite (HDM) allergens are among the most clinically significant allergens affecting humans. The medical outcomes upon exposure to HDM include the development and exacerbation of asthma, atopic dermatitis and allergic rhinitis. In humans, the group I antigens of *Dermatophagoides pteronyssinus* (Der p 1) are among the most common aeroallergens associated with atopic asthma [8]. Der p 1 is a cysteine protease, which degrades tight junctions in the respiratory epithelium and cleaves CD23 from activated B cells and CD25 from T cells [9]. Through its protease activity, it may therefore have a direct impact on immune polarization and act as a Th2 adjuvant in allergic inflammation.

In mice, BCG has been shown to suppress allergen-induced airway eosinophilia, a hallmark of allergic asthma, and airway hyper-responsiveness to metacholine [10]. This is accompanied by a decrease in Th-2-type cytokine production, such as IL-5, in broncho-alveolar lavage fluids, shifting the immune balance towards a Th-1 profile. Independent studies have confirmed these observations and have suggested a mechanism involving BCG-induced upregulation of IL-12 and IFN-γ production by innate immune cells [1]. We have previously shown that administration of recombinant BCG producing murine IL-18 (rBCG-mIL-18), which acts in synergy with IL-12 to induce IFN-γ production by T cells, further increases Th-1 cytokine production in mice [11]. Furthermore, this strain was found to protect better against allergen-induced eosinophilia and IL-5 production than non-recombinant BCG in a murine model of experimental ovalbumin-induced asthma [12]. Subsequently, we have shown that compared to non-recombinant BCG, a recombinant BCG strain producing human IL-18 (rBCG-hIL-18) also induces stronger IFN-γ production by T cells cocultured with dendritic cells (DCs) from human volunteers vaccinated with BCG [13]. However, it is not known whether BCG and rBCG-hIL-18 have differential effects on DCs and T cells of allergic patients compared to healthy subjects. 

Since DCs play a pivotal role in the orientation of T cells and the development of allergic responses [14], we compared here the DC phenotype and cytokine production of allergic patients with those of healthy donors upon stimulation with BCG or rBCG-hIL-18 in the presence or absence of Der p 1. We also compared the naïve and memory T cell responses of allergic patients with those of healthy donors cocultured with BCG- or rBCG-hIL-18-stimulated DC in the presence of Der p 1.

## 2. Materials and Methods

### 2.1. Ethics Statement

The study was conducted according to the principles of the Declaration of Helsinki and was approved by the Ethics Committee of the Medical University in Lodz, Poland (number RNN/169/08/KE). Written informed consent from all study subjects was obtained before blood sampling.

### 2.2. Human Donors

Blood was collected from twenty-two 19–35 year old allergic patients, sensitive to Der p 1, and from 40 healthy non-allergic, non-asthmatic subjects. The healthy subjects were enrolled at the Faculty of Biology and Environmental Protection, University of Lodz, Poland. Patients were recruited from the University Hospital Allergy Clinic, Department of Immunology and Allergy, Medical University of Lodz. Allergic patients had a clinician’s diagnosis of asthma and presented the features of HDM sensitization, including positive skin prick tests (SPTs), and/or the presence of HDM-specific IgE in serum. SPTs were performed as detailed by the European Academy of Allergy and Clinical Immunology [15,16]. All allergic donors refrained for 4 days before blood collection from oral antihistaminic drugs or leukotriene receptor antagonists but were maintained on routine inhaled corticosteroids. No patient was undergoing allergen-specific immunotherapy or took oral corticosteroids at the time of the study. Healthy subjects did not display any allergic characteristics and had negative SPTs. Their total IgE levels were less than 20 kU/L, and they had no specific serum IgE to *D. pteronyssinus* (<0.35 kU/L). All volunteers and allergic patients had received neonatal vaccination with BCG Moreau (2–5 doses). The subjects’ characteristics are shown in Table S1. Peripheral blood was drawn using vacutainer tubes with spray-coated heparin (Becton Dickinson) to obtain CD14^+^ monocytes and CD4^+^ T cells. 

### 2.3. Human Monocyte-Derived Dendritic Cell (MD-DC) Preparation 

The MD-DCs were generated from peripheral mononuclear cells (PBMCs), prepared from blood collected on heparin, by positive selection using monoclonal anti-CD14 antibodies coupled to magnetic microbeads (Miltenyi Biotech, Bergisch Gladbach, Germany), as described by Pochard et al. [17]. Cells were cultured at 1 × 10^6^ cells/mL for 6 days in complete medium containing 25 ng/mL human granulocyte–macrophage colony stimulating factor (GM-CSF) and 10 ng/mL human recombinant IL-4 (R&D Systems, Minneapolis, MN, USA) to obtain immature DCs. After 6 days of culture, the cells were harvested, pooled and counted. The mean percentages of DCs obtained from monocytes isolated from allergic patients and healthy donors were 35.4 ± 3.4 and 31.7 ± 5.2, respectively (*p* < 0.05).

### 2.4. Preparation of Bacteria

*M. bovis* BCG strain 1173P2 (WHO Stockholm, Stockholm, Sweden) and rBCG-hIL-18 were prepared as described [13]. Briefly, the mycobacteria were grown to the mid-log phase in stationary flasks at 37 °C in 7H9 Middlebrook liquid medium (Becton Dickinson, Warsaw, Poland) supplemented with 10% oleic acid–albumin–dextrose catalase (OADC) (Difco, BD Biosciences, Warsaw, Poland) and 0.05% Tween 80. For rBCG-hIL-18, 20 μg/mL kanamycin was added to the culture. Production of IL-18 was verified by immunoblotting as described [13]. The bacteria were serially diluted in PBS + 0.05% Tween 80 and plated onto Middlebrook 7H11 agar supplemented with 10% OADC and 20 μg/mL kanamycin where appropriate. After 3–4 weeks at 37 °C, colony-forming units were counted.

### 2.5. Dendritic Cell Activation

Immature DCs at a density of 1 × 10^6^ cells/mL were incubated for 24 h at 37 °C and 5% CO_2_ with live BCG or rBCG-hIL-18 at a multiplicity of infection of 1, according our previously established protocol [18]. Der p 1, kindly provided by G.A. Stewart (University of Western Australia) and Joël Pestel (Institut Pasteur de Lille), was used at 1 μg/mL [18,19]. This concentration had previously been determined as being optimal for the induction of Th-2 cytokines in allergic patients [17]. As positive maturation controls, DCs were stimulated with 1 μg/mL LPS (*Escherichia coli* LPS O55:B5, Sigma-Aldrich Chemical), while DCs in medium alone represented the negative controls. Supernatants of the cultures were tested by ELISA (Eli-pair Diaclone test) for the presence of IL-10, IL-12p70 and IL-23 (detection sensitivity: 5 pg/mL for IL-10 and IL-12p70; 20 pg/mL for IL-23), IP-10 (CXCL10) (R&D systems; detection sensitivity: 5 pg/mL), TARC (CCL17) and MDC (CCL22) (R&D systems; detection sensitivity: 10 pg/mL).

### 2.6. Dendritic Cell Surface Marker Analysis

The stimulated and unstimulated MD-DCs were harvested from the 6-well plates using PBS/2 mM EDTA. They were then washed in PBS and stained for 30 min at 4 °C with the following monoclonal antibodies (mAbs, Becton Dickinson, Warsaw, Poland): fluorescein isothiocyanate (FITC)-conjugated anti-CD86, anti-CD40, anti-HLA-DR, anti-CD40 and anti-DC-SIGN mAbs; phycoerythrin (PE)-conjugated anti-CD80 mAb; or relevant isotype-matched mAbs as controls. Living cells were gated using forward and side scatter properties (FSC/SSC) and then using the specific markers indicated above. Data were analyzed using the FACS LSRII (BD) and FlowJo software. Compensations were calculated using BD Compbeads with the automatic compensation program. Data were expressed as percentages of cells expressing the marker and the mean fluorescence intensities (MFI), representing the molecular densities on the cell surface for each marker for the considered population, after subtraction of the isotype control.

### 2.7. Naive and Memory CD4^+^ T Cell Isolation

Naïve CD45RA^+^CD4^+^ T cells and memory CD45RO^+^CD4^+^ T cells were isolated from the eluted CD14^–^ cell fraction using a naïve CD4^+^ T-cell isolation kit and a memory CD4^+^ T cell isolation kit (Miltenyi Biotec), respectively, as described [18]. Both cell fractions (purity > 95%) were frozen at −80 °C in FCS containing 10% DMSO until used.

### 2.8. DC-T Cell Cocultures

The frozen naive and memory T lymphocytes at 1 × 10^7^ cells/mL were thawed and cocultured for 96 h at 37 °C and 5% CO_2_ with BCG- or rBCG-hIL-18-primed autologous DCs, in the presence or absence of 1 µg/mL Der p 1, at a ratio of 10 lymphocytes per stimulated DC. Collected supernatants were tested for IFN-γ, IL-10 and IL-5 secretion by ELISA using the Diaclone kit. The limit of detection was 5 pg/mL for all three cytokines.

### 2.9. Statistical Analysis 

Statistical significance of differences was determined by using the Statistica 10.0 PL software (Statsoft). After verifying assumptions, including normality by using the Kolmogorov–Smirnov test, homogeneity of variance with the Levene test, the type of data and the number of data, the non-parametric tests were used. The Kruskal–Wallis test was used to determine the differences between stimulation conditions. When statistical significance was observed, differences were analyzed by the non-parametric Mann–Whitney U test for unpaired data. *p* values < 0.05 were considered significant.

## 3. Results

### 3.1. rBCG-hIL-18 and BCG Induce the Maturation of MD-DCs in the Presence of Der p 1

**Figure 1:**
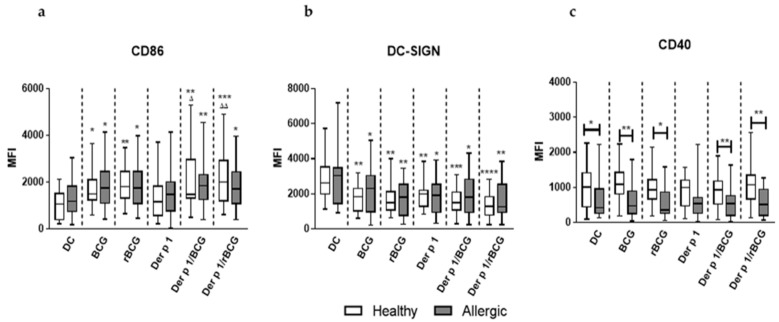
CD86 (**a**), DC-SIGN (**b**) and CD40 (**c**) surface expression on MD-DCs from healthy donors and allergic patients. Human MD-DCs were stimulated either with BCG (1:1), rBCG-hIL-18 (rBCG) (1:1), Der p 1 (1 μg/mL), Derp1/BCG or Der p 1/rBCG-hIL-18 for 24 h or were left unstimulated (DC). Box plots represent the median MFI and interquartile range values for 22 allergic donors and 40 healthy donors. Fluorescence intensity was calculated by the MFI (once for each donor) of the receptor expression from which the MFI obtained with an isotype-matched antibody was subtracted. Statistical analyses were performed by using the Kruskal–Wallis non-parametric test. * *p* < 0.05; ** *p* < 0.01; *** *p* < 0.001; **** *p* < 0.0001; versus control (unstimulated DC), unless indicated by the horizontal bars; ^Δ^ *p* < 0.05; ^ΔΔ^ *p* < 0.01 versus Der p 1.

During maturation, DCs coordinately regulate antigen capturing, processing and presentation, as well as the expression of co-stimulatory molecules and cytokine production [20]. To examine whether the allergic status affects the maturation of MD-DCs induced in vitro with BCG or rBCG-hIL-18 in the presence of Der p 1, we analyzed by flow cytometry the DC-surface expression of various co-stimulatory molecules and surface receptors that play an important role in the DC–T cell interaction. MD-DCs from allergic patients and healthy donors were pulsed with BCG or rBCG-hIL-18, alone or in the presence of Der p 1. The characteristics of blood donors are presented in Table S1. A total of 10,000 events were collected in the gates. The MFI of CD86, CD80, HLA-DR, CD40 and DC-SIGN, as well as the percentages of the cells gated on the FSC/SSC spectrum, were determined for allergic patients and healthy donors. The percentages of gated cells were similar in each experiment, and the values ranged from 82 to 90%. Both for allergic patients and healthy donors, BCG and rBCG-hIL-18, with or without (+/−) Der p 1, significantly upregulated the expression of CD86 compared with unstimulated DCs or DC incubated with Der p 1 alone (Figure 1a). In parallel, a significant decrease in DC-SIGN expression was observed upon incubation with BCG or rBCG-hIL-18, +/− Der p 1 compared to unstimulated DCs in allergic patients and in healthy donors (Figure 1b). No significant differences between healthy controls and allergic patients were noted for CD86 and DC-SIGN expression. This was unlike CD40, which was significantly less expressed on the surface of DCs from allergic patents compared to healthy controls, regardless of the presence of either BCG strain (Figure 1c). No significant alternation in CD80 and HLA-DR expression on MD-DCs stimulated with mycobacteria alone or in the presence of Der p 1 was found in either group of donors. (Figure S1). There was no difference between BCG- and rBCG-hIL-18-stimulated cells for any of these markers. 

### 3.2. Effect of BCG and rBCG-hIL-18 on Cytokine and Chemokine Production by DCs in the Presence of Der p 1

**Figure 2:**
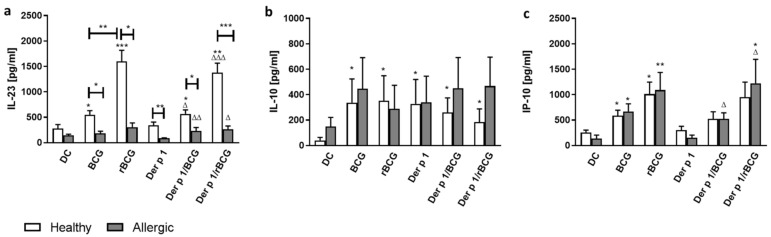
IL-23 (**a**), IL-10 (**b**) and IP-10 (**c**) production by stimulated MD-DCs from allergic patients and healthy donors. Human MD-DCs were stimulated with BCG (1:1), rBCG-hIL-18 (rBCG) (1:1), Der p 1 (1 μg/mL), Der p 1/BCG or Der p 1/rBCG for 24 h or were left unstimulated (DC). The cytokine (IL-23, IL-10) and chemokine (IP-10) levels in culture supernatants were measured in duplicate by ELISA. Data shown represent the medians ± SEM for 22 allergic patients and 40 healthy donors. Statistical analyses were performed by using the Mann–Whitney U test for unpaired data. * *p* < 0.05; ** *p* < 0.01; *** *p* < 0.001 versus control (unstimulated DC), unless indicated by the horizontal bars, ^Δ^ *p* < 0.05; ^ΔΔ^ *p* < 0.01; ^ΔΔΔ^ *p* < 0.001 versus Der p 1.

As in mice, BCG and IL-18-producing recombinant BCG are strong Th1-inducers [11] and may reduce Th2-dependent inflammatory processes [10,12]. We measured the concentrations of IL-12p70, IL-23, IL-10 and IP-10 in the supernatants of the MD-DCs incubated with BCG or rBCG-hIL-18 +/− Der p 1. While for all blood donors, LPS-stimulated MD-DCs produced significant levels of IL-12p70, BCG- and rBCG-hIL-18-pulsed MD-DCs +/− Der p 1 failed to secrete significant amounts of IL-12p70 in healthy donors and allergic patients (data not shown). In contrast to IL-12p70, the MD-DCs from healthy donors incubated with BCG or rBCG-hIL-18 +/− Der p 1 resulted in significant IL-23 production compared to non-pulsed DCs. This was not seen for allergic patients. Baseline IL-23 production, in the absence of BCG or rBCG-hIL-18 +/− Der p 1, was also lower in allergic patients than in healthy controls. Moreover, the rBCG-hIL-18-pulsed MD-DCs from healthy donors produced significantly more IL-23 than BCG-pulsed MD-DCs (Figure 2a). 

MD-DCs from healthy donors incubated with BCG or rBCG-hIL-18 +/− Der p 1 also produced significantly more IL-10 compared to unstimulated cells (Figure 2b). This trend was also seen for the allergic patients but did not reach statistical difference. There was no difference between BCG and rBCG-hIL-18. Furthermore, for allergic patients and for healthy donors, BCG and rBCG-hIL-18 increased the IP-10 (CXCL10) production by MD-DCs, compared to unstimulated MD-DCs, in both the presence and absence of Der p 1. This was particularly striking and significant with rBCG-hIL-18 (Figure 2c). Finally, since CCL17/TARC and CCL22/MDC are associated with the induction of chemotaxis in Th2 cells, we also measured these chemokines produced by DCs from allergic patients and healthy controls cultured in the different conditions. TARC and MDC production was significantly increased in both allergic patients and in the controls after incubation of DCs with Der p 1, and there was a trend toward a decrease in the production of these cytokines when the DCs were incubated in addition with BCG or rBCG-hIL-18, which reached statistical significance for the allergic patient rBCG-hIL-18 group (Figure S2).

### 3.3. IFN-γ Production by CD4^+^ Naive and Memory T Cells Cocultured with Der p 1-Treated DCs in the Presence of BCG or rBCG-hIL-18 

**Figure 3:**
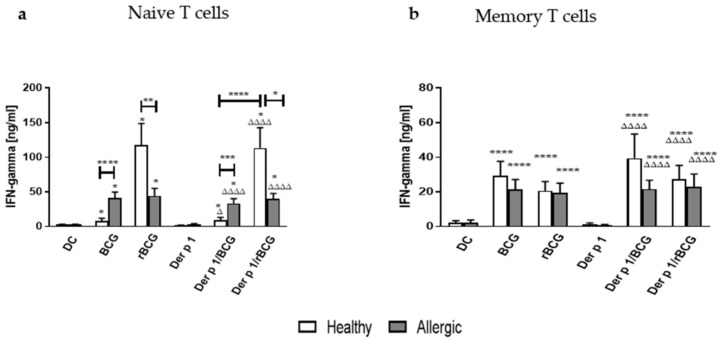
Secretion of IFN-γ by human naive T cells (**a**) and memory T cells (**b**) following 96 h coculture with BCG- (1:1), rBCG-hIL-18 (rBCG)- (1:1), Der p 1- (1 μg/mL), Der p 1/BCG- or Der p 1/rBCG-pulsed autologous MD-DCs (ratio MD-DCs/T cells, 1:10). The cytokine levels in the cocultures were measured in duplicate by ELISA. Data shown are the medians ± SEM for 22 allergic patients and 40 healthy donors. Statistical analyses were performed by using the Kruskal–Wallis test for unpaired data. * *p* < 0.05; ** *p* < 0.01; *** *p* < 0.001; **** *p* < 0.0001 versus control (unstimulated DC), unless indicated by the horizontal bars; ^Δ^ *p* < 0.05; ^ΔΔΔΔ^ *p* < 0.0001 versus Der p 1.

To investigate whether BCG- or rBCG-hIL-18-pulsed DCs can polarize T cells towards the Th1 profile, despite the presence of Der p 1, in patients with asthma, we measured the Th1-type cytokine (IFN-γ) levels in the supernatants collected from DC-autologous naive and DC-autologous memory T cell 96-h cocultures (Figure 3). Naive T cells produced significantly more IFN-γ in response to BCG- or rBCG-hIL-18-pulsed DCs +/− Der p 1, as compared to T cells incubated with non-pulsed or Der p 1-pulsed DCs, in allergic patients and in healthy donors (Figure 3a). However, in response to BCG- and Der p 1/BCG-pulsed DCs, naive T cells from allergic patients produced significantly more IFN-γ than those from healthy donors, while the reverse was observed for rBCG-hIL-18- and for Der p 1/rBCG-hIL-18-pulsed DCs co-incubated with naive T cells. Strikingly, the IFN-γ production of naive T cells from healthy donors was only marginally enhanced by BCG-pulsed DCs but was much more strongly induced when the T cells were incubated with rBCG-hIL-18-pulsed DCs. For the allergic patients, there was no difference between the BCG and rBCG-hIL-18 groups, regardless of coincubation of the DCs with Der p 1.

Coincubation of BCG- or rBCG-hIL-18-pulsed DCs +/− Der p 1 also induced the IFN-γ production by memory T cells in allergic patients and healthy donors. However, in this case there was no significant difference in the IFN-γ production between allergic patients and healthy donors, nor was there a difference between BCG and rBCG-hIL-18, regardless of the presence of Der p 1 (Figure 3b). 

### 3.4. Effect of BCG and rBCG-hIL-18 on IL-5 and IL-10 Production by CD4^+^ Naive and Memory T Cells Cocultured with Der p 1-Treated DCs 

**Figure 4:**
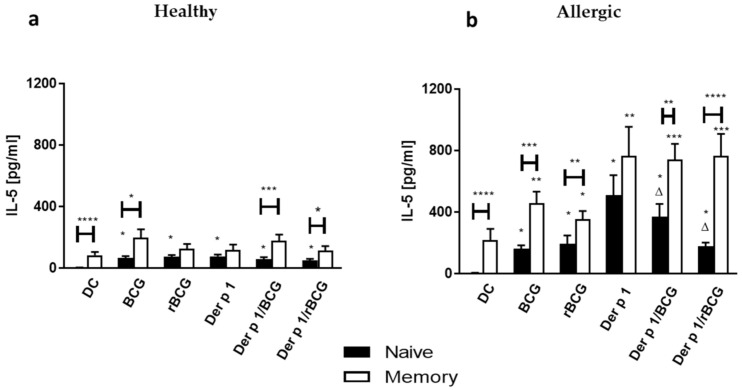
Secretion of IL-5 by human naive T cells (**a**) and memory T cells (**b**) following 96 h coculture with BCG- (1:1), rBCG-hIL-18 (rBCG)- (1:1), Der p 1- (1 μg/mL), Der p 1/BCG- or Der p 1/rBCG-pulsed autologous MD-DCs (ratio MD-DCs/T cells, 1:10). The cytokine levels in the cocultures were measured in duplicate by ELISA. Data shown are the medians ± SEM for 22 allergic patients and 40 healthy donors. Statistical analyses were performed by using the Kruskal–Wallis test for unpaired data. * *p* < 0.05; ** *p* < 0.01; *** *p* < 0.001; **** *p* < 0.0001 versus control (unstimulated DC), unless indicated by the horizontal bars; ^Δ^ *p* < 0.05 versus Der p 1.

As Th2 responses are considered a hallmark of allergic pulmonary inflammation, we compared the IL-5 production by autologous CD4^+^ naive and memory T cells upon coincubation with DCs conditioned with BCG or rBCG-hIL-18 +/− Der p 1 between allergic patients and healthy donors. Naive T cells from healthy donors, but more so from allergic patients, produced significantly more IL-5 in response to BCG- or rBCG-hIL-18-stimulated DCs than unstimulated DCs (Figure 4a). The strongest IL-5 response was seen after co-culturing naive T cells with Der p 1-conditioned DCs from allergic patients. This response was significantly lower for the healthy controls. Interestingly, BCG, and especially rBCG-hIL-18, substantially diminished the IL-5 responses of the naive T cells from allergic patients. The Der p 1 effect was totally abolished by rBCG-hIL-18. The BCG and rBCG-hIL-18 effects in the presence of Der p 1 were not seen for the healthy donors. BCG- or rBCG-hIL-18-stimulated DC, especially in the presence of Der p 1, also induced the IL-5 production of memory T cells form allergic patients, but not from healthy controls (Figure 4b). Again, coincubation of memory T cells with Der p 1-pulsed DCs induced the strongest IL-5 response. In this case the IL-5 response was not diminished by BCG or rBCG-hIL-18.

**Figure 5:**
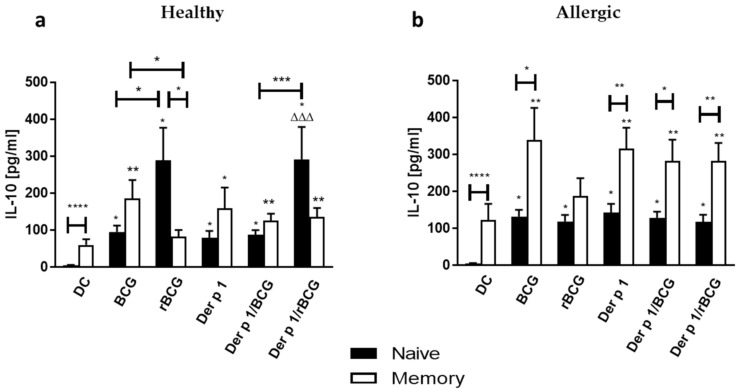
Secretion of IL-10 by human naive T cells (**a**) and memory T cells (**b**) following 96 h coculture with BCG- (1:1), rBCG-hIL-18 (rBCG)- (1:1), Der p 1- (1 μg/mL), Der p 1/BCG- or Der p 1/rBCG-pulsed autologous MD-DCs (ratio MD-DCs/T cells, 1:10). The cytokine levels in the cocultures were measured in duplicate by ELISA. Data shown are the medians ± SEM for 22 allergic patients and 40 healthy donors. Statistical analyses were performed by using the Kruskal–Wallis test for unpaired data. * *p* < 0.05; ** *p* < 0.01; *** *p* < 0.001; **** *p* < 0.0001 versus control (unstimulated DC), unless indicated by the horizontal bars; ^ΔΔΔ^ *p* < 0.001 versus Der p 1.

The IL-10 production by naive T cells co-incubated with BCG- or rBCG-hIL-18-pulsed DCs +/− Der p 1 was also enhanced for allergic patients and for healthy controls. Coincubation of naive T cells with rBCG-hIL-18-pulsed DCs from healthy donors induced significantly more IL-10 than coincubation with BCG-pulsed DCs, regardless of the addition of Der p 1. This was not the case for allergic patients, for which neither BCG nor rBCG-hIL-18 modified the IL-10 response of naive T cells cocultured with Der p 1-pulsed DCs (Figure 5a). The IL-10 production by memory T cells from healthy donors was only marginally increased by coincubation with pulsed DCs. This increase was somewhat stronger for memory T cells from allergic patients, especially when they were cocultured with DCs pulsed with Der p 1 (Figure 5b). Like for the naive T cells, BCG or rBCG-hIL-18 did not modify the IL-10 response of memory T cells co-cultured with Der p 1-pulsed DCs.

## 4. Discussion

In order to explore the immunomodulatory effects of BCG and rBCG-hIL-18 in a Der p 1-induced allergic environment, we used a well-established in vitro model of DC-T cell cocultures, as professional antigen-presenting cells DCs play a key role in the initiation and development of allergic diseases. Although accumulating evidence suggests that DCs can be sufficient to initiate Th2 responses, the signaling mechanism between DCs and T cells underlying the Th2 profile is still not well understood [21]. Immune responses in allergic individuals are characterized by excessive production of Th2-related cytokines by allergen-specific CD4^+^ T cells and a lower Th1-type immune response than healthy subjects [3,4]. BCG is known to induce Th1 lymphocytes [10,22,23], suggesting that BCG could contribute to asthma prevention. In this study, we demonstrate in a model of HDM allergy that in the presence of Der p 1, the IL-5 production by naïve T cells from allergic patients was significantly decreased by BCG and even more by rBCG-hIL-18. This was not the case for healthy subjects. Memory T cells also produced IL-5 in response to Der p 1-pulsed DCs, but this was not decreased by BCG or rBCG-hIL-18. These observations are consistent with previous studies showing a decrease of Th2 cytokines in Der p 1-pulsed DC-T cell cocultures from allergic patients when BCG was used together with a *D. farinae* extract [24]. However, in this previous study no distinction was made between naive and memory T cells. In a murine model, BCG or rBCG-mIL-18, when administrated at the time of sensitization, prevented airway responses after ovalbumin challenge [12]. 

As memory T cells of allergic patients have already been primed by the antigen, their triggering requirements are different from those of naive T cells. Memory T cells are more agile and migratory than naive T cells, which is consistent with their prime mission to survey tissues for pathogen antigens. Naive and memory T cells express different sets of chemokine receptors and cell adhesion molecules [25]. Naive T cells express large amounts of the chemokine receptor CCR7 and the cell adhesion molecule CD62L, which facilitate their migration and entrance into secondary lymphoid tissues. On the other hand, memory T cells express CCR9 and CXCR3, which promote trafficking to peripheral tissues. Furthermore, memory T cells, but not naive T cells, preferentially home to the bone marrow, where they undergo expansion and homeostatic proliferation [26,27]. In addition to conventional Th2 memory T cells, a new subset of human proallergic memory Th2 cells, named Th2A (CD4^+^CD27^−^CD45RB^−^), has been identified [28]. This subset is confined to allergic individuals and exhibits distinct features. It was identified in allergic donors with different types of allergy, including allergy to HDM. It was also found to play a critical role in allergy pathogenesis. As in this study we have not examined the subtypes of memory T cells, we cannot exclude that BCG or rBCG-hIL-18 may have an effect on IL-5 production in one of the subtypes. However, if BCG or rBCG-hIL-18 would have an effect on IL-5 secretion by one of the memory T cell subsets, one would expect to see at least a trend toward a decrease of IL-5 secretion, which we did not observe. 

Differences between naive and memory T cell responses to BCG or rBCG-hIL-18 have been noted before in healthy donors with respect to secretion of other cytokines, namely IFN-γ and IL-10. Both were increased, especially by rBCG-hIL-18 [18]. IL-18 together with IL-7 was shown to synergistically upregulate the expression of IL-18R genes in naive T cells but not in memory T cells, thereby enhancing IL-18 activity in naive T cells [29]. Naive T cells depend on IL-7 for survival and homeostatic proliferation [30]. Furthermore, naive and memory T cells have different survival requirements for cytokines, including IL-7 [31]. The differences in cytokine requirements and IL-18R upregulation between naive and memory T cells may at least partly explain why naïve T cells from allergic patients responded to BCG and even more to rBCG-hIL-18 by decreasing IL-5 production, whereas memory T cells did not.

IL-18, initially referred to as interferon IFN-γ-inducing factor, was first described for its ability to induce Th1 responses, resulting in the production of IFN-γ [32] and subsequently inhibiting Th2 responses [33,34,35,36]. However, in a ragweed murine model of allergic asthma, administration of IL-18 together with the allergen increased the production of IL-5 by splenocytes cultured in the presence of ragweed. This suggests that IL-18 can promote a Th2 phenotype [37], which is in contradiction to our observations. On the other hand, when IL-12 was co-administered with IL-18 in a murine allergic asthma model, the appearance of Th2 cytokines was abolished. This was paralleled by the induction of Th1 cytokines, suggesting a synergistic effect of IL-12 and IL-18 in the prevention of Th2-cell differentiation [35]. BCG is known to induce IL-12 production [38]. Therefore, it is likely that in our model the IL-18 effect is the result of the recombinant IL-18 and IL-12 naturally induced by the BCG. Interestingly, the IL-5 response to Der p 1 in allergic patients already significantly decreased with non-recombinant BCG. However, the decrease was stronger in the presence of rBCG-hIL-18, consistent with the synergistic effect of BCG and IL-18.

In parallel, BCG and rBCG-hIL-18 increased the production of the Th1 cytokine IFN-γ by naïve and by memory T cells. Again, a synergistic effect of BCG and IL-18 was observed for the IFN-γ production, but only in naïve T cells from healthy donors, as we have seen in a previous study [13]. This observation is also consistent with previous studies in mice showing that in response to rBCG-mIL-18 Th1, cytokine production was favored in a synergistic manner [12]. Here, we found that the effect of BCG and rBCG-hIL-18 on IFN-γ production was observed for naïve and for memory T cells of healthy subjects and of allergic patients. This was in contrast to the effect of BCG and rBCG-hIL-18 on the decrease of IL-5 production, which was limited to the naive T cells from allergic donors. However, the synergistic effect of IL-18 and BCG on IFN-γ production was only observed for naive T cells from healthy subjects. Thus, the IL-5 decrease observed in naive T cells from allergic patients after BCG- or rBCG-hIL-18-pulsed DC treatment was not directly linked to the IFN-γ production. 

We did not see a significant induction of IL-12p70 production by BCG- or rBCG-hIL-18-pulsed MD-DCs +/− Der p 1. IL-12 is a heterodimeric cytokine composed of subunits p40 and p35, and the p40 subunit is shared with IL-23. We therefore determined the amount of secreted IL-23 in the supernatants of mycobacteria-stimulated DCs +/− Der p 1. We found that MD-DCs from healthy donors incubated with BCG or rBCG-hIL-18 +/− Der p 1 induced significant amounts of IL-23. This was not seen for the MD-DCs from allergic patients. Furthermore, a synergistic effect of IL-18 and BCG was observed for the healthy donors. Several murine studies have suggested a pathogenic role of IL-23 in allergic airway inflammation [39,40]. In an allergic model of murine asthma, IL-23 produced by DCs at the site of antigen sensitization facilitated eosinophilia and Th2 immune responses [41]. A potential role of IL-23 increase in asthma pathogenesis has also been proposed in the context of Der p 1 exposure [42]. In humans, a significant relationship between serum levels of IL-23 and its gene expression and persistent asthma in children was suggested to be a biomarker of asthma [43]. Curiously, we found that IL-23 production, in the absence of BCG or rBCG-hIL-18, but in the presence of Der p 1 was lower in allergic patients than in healthy controls. BCG or rBCG-hIL-18 did not decrease IL-23 expression any further in the MD-DCs of asthma patients. A tendency of decreased IL-23 production by MD-DCs was already observed in unstimulated DCs from allergic patients compared to unstimulated DCs from healthy subjects. Therefore, in our study the BCG- or rBCG-hIL-18-mediated decrease of IL-5 production by naive T cells from allergic patients was not directly linked with the level of IL-23 production by MD-DCs. We did see a trend toward a decrease in the chemokine TARC and MDC production by BCG- or rBCG-hIL-18-treated DCs from allergic patients in the presence of Der p 1. This decrease was slight but significant for the rBCG-hIL-18-treated DCs from the allergic patients. As these chemokines are known to direct Th2 responses [44], the effect of BCG-, and especially rBCG-hIL-18-treated DCs, on the decrease of IL-5 production by naïve T cells from allergic donors may be related to the decrease in production of these chemokines. 

IP-10 production by MD-DCs was also increased by BCG and rBCG-hIL-18 +/− Der p 1. This was observed for healthy donors, as previously reported [13], and for allergic patients, as shown here. For both, rBCG-hIL-18 had a tendency to induce stronger IP-10 secretion than non-recombinant BCG, although this difference did not reach statistical significance. IP-10 belongs to the CXC chemokine subfamily and interacts with the common receptor CXCR3 that is highly expressed on Th0, Th1 and NK lymphocytes and to a lesser extent on eosinophils. IP-10 plays an important role in Th1-mediated immune responses, and IP-10 produced by mycobacteria-stimulated DCs is involved in the attraction of Th1 cells [45,46]. Some human studies suggest that IP-10 is a marker of asthma exacerbation [47,48,49]. However, other studies show low serum IP-10 levels [50,51] and a decreased production of IP-10 by PBMCs activated by mitogen, allergen or cytokines in asthmatic patients compared to healthy controls [51]. Similarly, in a murine model of asthma induced by *Alternaria*, the production of poly I:C-induced IP-10 by DCs was significantly reduced by incubation with the fungal extract [52]. In our study, we found that in allergic patients, increased IP-10 production by DCs upon stimulation with BCG or rBCG-hIL-18 was paralleled with decreased production of IL-5 by naive T cells when cocultured with Der p 1-stimulated DCs, but not with unstimulated DCs. No relationship was found between IP-10 production by mycobacteria-pulsed DCs and IL-5 production by naïve T cells from healthy donors, regardless of Der p 1 stimulation. These observations suggest that IP-10 may exert its effect on antigen-specific T cells in asthmatic patients.

In contrast to IP-10, IL-10 production by DCs was not strongly affected by BCG or rBCG-hIL-18 in the context of Der p 1 stimulation, neither for healthy controls, nor for asthmatic patients. Der p 1 alone appeared to stimulate IL-10 production by DCs from both groups, which was not modified by either BCG strain. However, in healthy controls, but not in allergic patients, rBCGh-IL-18-pulsed DCs induced significantly increased IL-10 expression by naive T cells compared to DCs pulsed with non-recombinant BCG. BCG- or rBCG-hIL-18-pulsed DCs had no effect on IL-10 production by memory T cells. Although IL-10 has been shown to prevent and even reverse the characteristic features of experimental asthma [53], we found no relationship between IL-10 production and decreased IL-5 production by naive T cells from asthmatic patients. 

By expressing specific costimulatory molecules, DCs play a crucial role in DC-T cell synapse signaling and can modulate the polarization of T cell responses. We found that both BCG and rBCG-hIL-18 stimulated the expression of CD86 on MD-DCs in allergic patients and in healthy controls. No difference with respect to CD86 expression was observed between these two groups. IL-18 did not appear to further enhance CD86 expression on MD-DCs. In contrast, DC-SIGN expression was decreased by BCG and rBCG-hIL-18 in both groups. However, this was also seen when the cells were stimulated with Der p 1 alone. BCG or rBCG-hIL-18 had no effect on the expression of CD80, CD40 or HLA-DR. There was a significant decrease in CD40 expression by MD-DCs in allergic patients compared to healthy controls, independently of Der p 1, BCG or rBCG-hIL-18 stimulation. Lowered expression of CD40 by DCs in allergic patients compared to healthy controls has been seen previously, albeit in a different model of human cockroach allergy [54]. Since CD40 plays an important role for IL-23 production by DCs [55], the lower CD40 surface expression by DCs from allergic patients compared to healthy controls is therefore consistent with a lower production of IL-23 by the DCs from allergic patients. However, this did not appear to have an impact on the observed BCG- and rBCG-hIL-18-mediated Der p 1 allergen-specific decrease in IL-5 production by naïve T cells from allergic patients.

## 5. Conclusions

We found that stimulation of DCs with BCG and more so with rBCG-hIL-18 in the presence of Der p 1 decreased IL-5 expression by naïve T cells from allergic patients but not by memory T cells. This effect was not related to an increase of the expression of costimulatory molecules on DCs but may be linked to an effect of the mycobacteria on the production of TARC and MDC by the DCs. As increased Th2-type IL-5 production is a hallmark of allergy, the use of BCG or, better yet, rBCG-hIL-18 may be a potential therapeutic tool to redress the Th1/Th2 immune balance in allergic patients.

## Data Availability

Data is contained within the article or Supplementary Materials.

## Supplementary Materials

The following are available online at https://www.mdpi.com/2076-393X/9/3/277/s1, Figure S1: CD80 and HLA-DR surface expression of MD-DCs from healthy donors and allergic patients; Figure S2: TARC and MDC production by stimulated MD-DCs from allergic patients and healthy donors; Table S1: Characteristics of blood donors included into the study.
