# Supplementary material for: Decrease of IL-5 Production by Naive T Cells Cocultured with IL-18-Producing BCG-Pulsed Dendritic Cells from Patients Allergic to House Dust Mite
Source: Vaccines (Basel). 2021 Mar 18;9(3):277. doi: 10.3390/vaccines9030277 (PMC8003153; doi:10.3390/vaccines9030277)
Supplement: Supplementary file 1 [file vaccines-09-00277-s001.zip › Table S1.pdf]

Table S1: Characteristics of blood donors included into the study

	Healthy donors	Asthmatic patients *p* values
Sex (F/M)	21/40 [60%]	6/22 [27.2%] *p*=0.055
Mean age (year)<br>range	26.72 ± 0.45<br>23-35	25.13 ± 1.18 *p*=0.141<br>19-35
Duration of asthma (y)	NA	12.8 ± 1.47
Atopy	0/40	22/22
Allergy to Der p 1	0/40	19/22
Treatment	None	Allergic donors refrained for 4 days before blood collection from oral antihistaminic drugs or leukotriene receptor agonists, but were maintained on routine inhaled corticosteroids, no patient was undergoing allergen-specific immunotherapy or took oral corticosteroid treatment at the time of the study
SPT [mm]	NA	3-12mm
Total IgE (kU/l)	<20	256.8 ± 48
IgE anti-Der p 1 (kU/l)	<0.35	18.9 ± 4.8

Data are presented as n or mean ± SEM. Y, year; SPT, Skin Prick test; NA, non applicable;, Der p 1, *Dermatophagoides pteronyssinus* allergen 1.
