# Supplementary figures and images for: Decrease of IL-5 Production by Naive T Cells Cocultured with IL-18-Producing BCG-Pulsed Dendritic Cells from Patients Allergic to House Dust Mite
Source: Vaccines (Basel). 2021 Mar 18;9(3):277. doi: 10.3390/vaccines9030277 (PMC8003153; doi:10.3390/vaccines9030277)

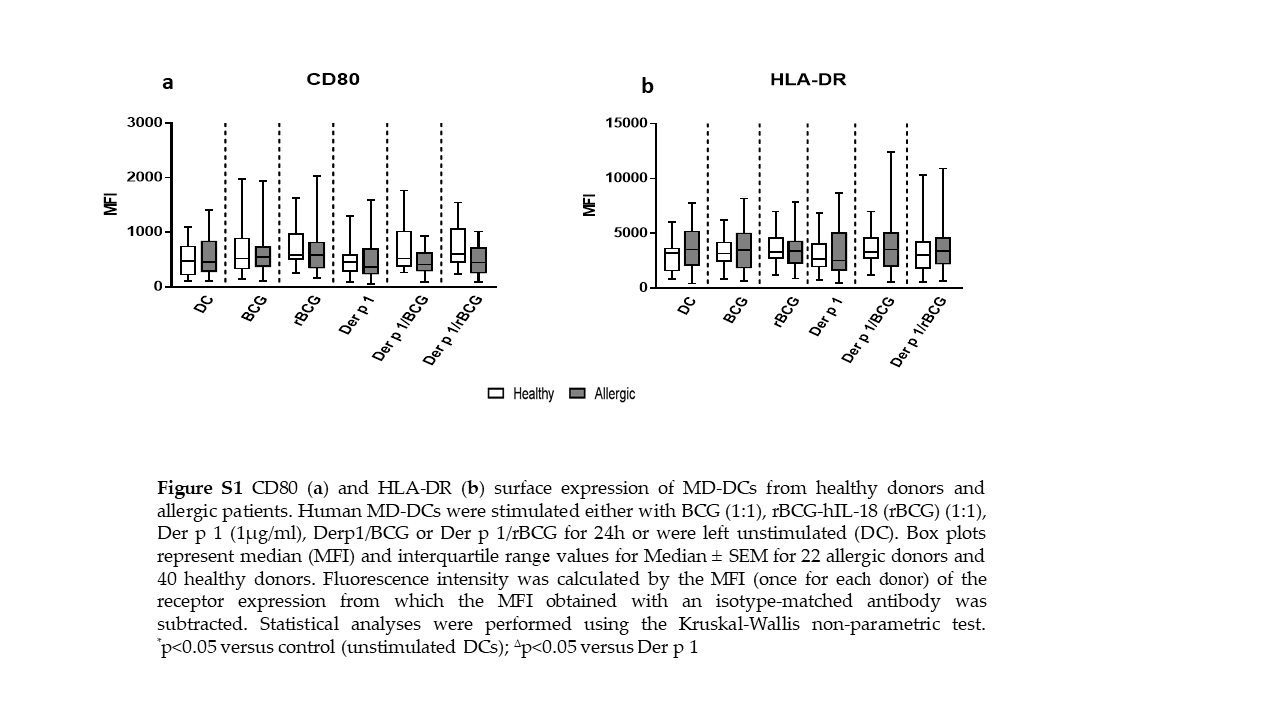

Supplement: Supplementary file 1 [file vaccines-09-00277-s001.zip › Figure S1 .tif]

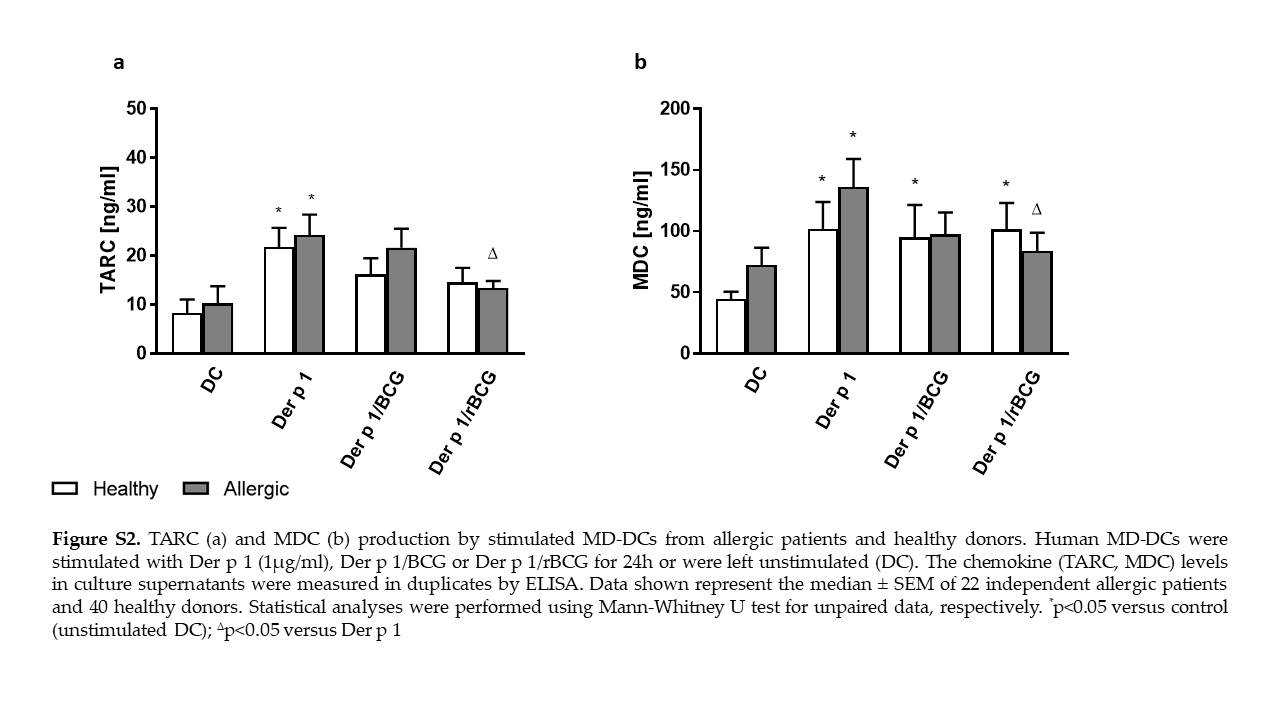

Supplement: Supplementary file 1 [file vaccines-09-00277-s001.zip › Figure S2 .tif]
